# Danegaptide Enhances Astrocyte Gap Junctional Coupling and Reduces Ischemic Reperfusion Brain Injury in Mice

**DOI:** 10.3390/biom10030353

**Published:** 2020-02-26

**Authors:** Moises Freitas-Andrade, John Bechberger, Jasmine Wang, Ken K.C. Yeung, Shawn N. Whitehead, Rie Shultz Hansen, Christian C. Naus

**Affiliations:** 1 Cellular & Physiological Sciences, Faculty of Medicine, Life Science Institute, University of British Columbia, Vancouver, BC V6T 1Z3, Canada; moisesfreitasandrade@gmail.com (M.F.-A.); jbechber@gmail.com (J.B.); 2 Anatomy & Cell Biology, Western University, London, ON N6A 5C1, Canada; jwang464@uwo.ca (J.W.); Shawn.Whitehead@schulich.uwo.ca (S.N.W.); 3 Zealand Pharma A/S, 2860 Copenhagen, Denmark; Rha@zealandpharma.com

**Keywords:** connexin43, stroke, gap junction, astrocytes, danegaptide

## Abstract

Ischemic stroke is a complex and devastating event characterized by cell death resulting from a transient or permanent arterial occlusion. Astrocytic connexin43 (Cx43) gap junction (GJ) proteins have been reported to impact neuronal survival in ischemic conditions. Consequently, Cx43 could be a potential target for therapeutic approaches to stroke. We examined the effect of danegaptide (ZP1609), an antiarrhythmic dipeptide that specifically enhances GJ conductance, in two different rodent stroke models. In this study, danegaptide increased astrocytic Cx43 coupling with no significant effects on Cx43 hemichannel activity, in vitro. Using matrix-assisted laser desorption ionization imaging mass spectrometry (MALDI IMS) the presence of danegaptide within brain tissue sections were detected one hour after reperfusion indicating successful transport of the dipeptide across the blood brain barrier. Furthermore, administration of danegaptide in a novel mouse brain ischemia/reperfusion model showed significant decrease in infarct volume. Taken together, this study provides evidence for the therapeutic potential of danegaptide in ischemia/reperfusion stroke.

## 1. Introduction

Stroke is a debilitating event associated with either mortality or significant reduction in quality of life. In ischemic stroke, occlusion of the middle cerebral artery (MCA) results in rapid loss of neurons in the infarct core while the peri-infarct, also referred to as the penumbra, is a region of instability that could be recovered [1]. Within the peri-infarct, reactive astrocytes expressing high levels of connexin43 (Cx43) in gap junctions (GJs) have been identified [2,3,4,5]. Several studies have shown that reduction of Cx43 levels is associated with detrimental outcome in ischemic stroke [6,7,8,9]. Collectively, these studies underscore Cx43 as a possible key player in brain ischemia and a potential target for therapeutic strategies.

In astrocytes, GJs are composed primarily of the channel protein Cx43 and, to a lesser extent, Cx30 and Cx26 [10,11,12]. Individual Cx43 proteins assemble into hexamers around a central hydrophilic pore to form transmembrane channels, termed connexons or hemichannels, which then couple with apposing connexons on neighboring cells forming a GJ intercellular channel. Individual GJs coalesce into dense plaques that may contain thousands of channels. However, hemichannels can also function as single cell membrane channels, particularly in pathological conditions [13]. GJ channels directly bridge the cytoplasm between coupled cells and permit the movement of ions and low molecular weight molecules (about 1–2 kDa) to neighboring cells or in the case of hemichannels, directly connect the cell cytoplasm to the extracellular milieu [13,14]. In the brain, astrocytic cell networks play an important role in homeostasis and repair after injury [15,16,17]. This coordinated interplay between astrocytes is achieved, in large part, through Cx43 channels.

Permanent middle cerebral artery occlusion (pMCAO) in transgenic cre/Cx43 floxed mice, where Cx43 is specifically deleted in astrocytes, resulted in significant increase in infarct volume, reduction in reactive astrocytes and an increase in apoptosis, 4 days after stroke insult [18]. Similarly, in Cx43 heterozygous null (Cx43^+/−^) mice, the loss of a single Cx43 allele in these mice resulted in reduced astrocyte coupling that led to increased neuronal death [6]. Interestingly, an in vitro study showed that Cx43 coupling between astrocytes delayed neuronal death in an oxygen/glucose deprivation model [19]. The authors concluded that astrocytic gap junctional networks provide protection against tissue damage during the acute phase of stroke [19]. This is consistent with the spatial buffering capacity associated with astrocytes [20] and may indicate the ability of Cx43-coupled astrocyte-networks to buffer or dissipate the toxic microenvironment affected by stroke.

Several lines of evidence demonstrate that inflammatory factors and hypoxia promote Cx43 hemichannel opening, resulting in both neuronal and astrocytic cell death [21,22,23,24,25]. We have recently shown that pharmacological inhibition of Cx43 hemichannels, without affecting Cx43 GJ coupling, is neuroprotective in stroke [26]. Similarly, Chen et al. (2019) showed neuroprotective effects by inhibiting Cx43 hemichannels in an ischemia/reperfusion mouse model [27]. However, whether increasing astrocyte Cx43 coupling in brain ischemia is neuroprotective is unknown.

Recent developments in Cx43 pharmacology have led to the synthesis of small molecules that can regulate Cx43 GJ coupling [28]. For example, the antiarrhythmic dipeptide ZP1609, referred to here as danegaptide, has been used to promote Cx43 GJ coupling in cardiomyocytes in myocardial infarction, resulting in reduced infarct size [29,30,31]. More recently, danegaptide was reported to prevent Cx43 GJ uncoupling and promote retinal vascular endothelial cell survival in diabetic retinopathy [32]. Whether danegaptide has a comparable beneficial effect in the ischemic brain is unknown.

The aim of this study was to determine whether danegaptide affects Cx43 function in astrocytes and if danegaptide improves stroke outcome in mice. We show that danegaptide significantly increases Cx43 GJ coupling in astrocytes while not affecting Cx43 hemichannel activity. Moreover, we demonstrate that danegaptide crosses the blood brain barrier (BBB) and administration after stroke/reperfusion is neuroprotective.

## 2. Materials and Methods

### 2.1. Permanent Middle Cerebral Artery Occlusion

All procedures were approved by the Animal Care Committee of the University of British Columbia. Wildtype (WT) C57Bl/6 mice from the Jackson laboratory (Bar Harbor, ME, USA) aged 3–4 months were used. Mice were maintained on a 12:12 h light:dark cycle, with food and water available ad libitum. Two different stroke models were performed: transient middle cerebral artery occlusion (tMACO) [33,34] and permanent middle cerebral artery occlusion (pMCAO) [18,35]. Briefly, the mice were anesthetized with sodium pentobarbital (65 mg/kg i.p.) and given an opiate (Buprenorphine, 0.1 mg/kg) and a local analgesic (Bupivacaine, 0.1 mL at 0.25%) at the incision site prior to surgery. Body temperature was maintained at 37 °C during surgery and recovery. For the tMCAO procedure, we used the tandem ipsilateral common carotid artery (CCA) middle cerebral artery (MCA) occlusion stroke model [33,34]. With the mouse in supine position a midline incision was made and the CCA was isolated and separated from the vagus nerve. The blood flow within the CCA was reduced with a 70-g pressure microclamp. The mouse was then rotated, and the head was held securely in place using a stereotaxic frame (David Kopf Instruments, Tujunga, CA, USA). With the aid of a dissecting microscope (Helmut Hund GmbH, Wetzlar, Germany), a skin incision was made on the right side of the head from the anterior of the ear towards the corner of the eye horizontally and from the corner of the eye vertically 5 mm. The squamosal bone was exposed by gently pulling back the temporal muscle. Using a fine battery-powered drill (Dremel, London, ON, Canada), a small hole was made ~2 mm in diameter on the skull bone to remove dura and expose the MCA. A metal pin was then placed under the MCA. The mouse was then placed in the supine position for temporary clamping of the CCA (Supplementary Figure S1). The CCA/MCA was blocked for 1 h. However, at 50 min, saline (pH 7.4) or 75 µg/kg of danegaptide (Zealand Pharma, Copenhagen, Denmark) was injected intravenously into the tail vein [30]. The 75 µg/kg dose was previously shown to be protective in pigs subjected to myocardial infarcts [30]. After the 1 h of ischemia, the clamp on the CCA and wire under the MCA were removed to allow reperfusion (Supplementary Figure S2). Cerebral blood flow was measured according to manufacturer’s protocol using the moorFLPI-2 system (Moor Instruments Inc, Wilmington, DE, USA). Mice were subsequently treated with 300 µg/kg of danegaptide by intraperitoneal (IP) injection at 1, 2, and 3 h after the initial injection. The larger 300 µg/kg concentration of danegaptide administered via IP is due to the fact that the absorption from this route is usually one-half to one-fourth as rapid as from the intravenous route [36]. Furthermore, the IP route was used in order to minimize animal stress and trauma due to repeated tail vein injection.

For the pMCAO procedure, exposure of the MCA was similar to the method described above and previously reported [18,37]. Briefly, once the MCA was exposed, the blood vessel was then cauterized above and below the rhinal fissure using an electronic coagulator (Codman and Shurtleff Inc., Raynham, MA, USA). After visually confirming the absence of reperfusion through the MCA, the skin incision was closed with sutures. Mice were then given IP injection of either saline or danegaptide (1–10 mg/kg) at 1, 2, 3 and 4 h post-pMCAO. Efforts were made to reduce the number of animals used and to minimize animal suffering.

Following injections of compounds, mice were allowed to recover for 48 h post-stroke. Mice were then anesthetized using a lethal dose of sodium pentobarbital (120/kg) IP and transcardially perfused with phosphate-buffered saline (PBS). The brains were quickly removed and rapidly frozen at −80 °C till further processing for infarct visualization by immunohistochemistry [37].

### 2.2. Quantification of Cerebral Infarction

A cryostat (HM 505E; Micron, Walldorf, Germany) was used to obtain 20 μm- and 10 μm-thick sections, collected at 200 µm intervals for infarct volume determination and immunohistochemistry. To prepare sections for measurement of infarct size, they were stained with 0.125% thionin (Fisher Scientific, Toronto, ON, Canada). Total infarct volumes were calculated using a stereological approach through the rostrocaudal extent of the infarct area, and corrected for edema as previously described [38]. Images were analyzed by an observer blind to experimental conditions with ImageJ software (Bethesda, MD, USA, available at: http://rsbweb.nih.gov/ij/).

### 2.3. Matrix-Assisted Laser Desorption/Ionization Imaging Mass Spectrometry (MALDI-IMS)

A CryoStar NX50 was used for all tissue sectioning. Tissue samples were mounted onto the tissue holder of the cryostat with water and coronal sections were obtained to 14 μm thicknesses at −25 °C. Each tissue section was approximately 5.5–9 mm by 7–9 mm. Once sliced, the sections were thaw mounted onto indium tin oxide (ITO) coated glass slides and placed in a desiccator for 10 min. A six-step wash first using, 70% ethanol, and 100% ethanol was employed. This was followed by a mixture of 60% ethanol, 30% chloroform, and 10% acetic acid. Finally, another 100% ethanol, water, and 100% ethanol was used. Upon tissue acidification, the water wash was replaced with 0.2% TFA. The tissue was incubated in this solvent at room temperature for 2.5 min.

2,5-dihydroxybenzoic acid (DHB) matrix was prepared to 20 mg/mL in 20% EtOH, 15% 100 mM ammonium citrate, and 1% phosphoric acid. DHB was applied onto sample sections using the automated TM sprayer from HTX Technologies. Complete sample coverage was obtained using 8 passes at a velocity of 1200 mm/min, and a flow rate of 0.05 mL/min. A final matrix density of 3.33 × 10^−2^ mg/mm^2^ was achieved. A rehydration step was performed using a 5% acetic acid solution. The sample slide was placed in the rehydration chamber at 70 °C for 3.5 min.

All MALDI-IMS sample analyses were performed on a Sciex TOF/TOF 5800 MALDI mass spectrometer (Toronto, ON, Canada). TOF/TOF Series Explorer in the positive ion, reflectron mode and Data Explorer were used for data acquisition and spectral processing, respectively. The Sciex TOF/TOF Imaging Acquisition Software (Toronto, ON, Canada) was used for region selection and image data acquisition. A total sum of 600 shots/spot and 30 shots/spot were acquired for spectral and imaging data acquisition, respectively, with a 1 kHz OptiBeam On-Axis Nd:YAG laser system (Toronto, ON, Canada). The open source MSiReader software (Toronto, ON, Canada) was used for imaging data manipulations and analysis.

### 2.4. Astrocyte Isolation and In Vitro Hypoxia

WT astrocytes were isolated from early postnatal (P0-P1) cortices as previously described [39]. Briefly, dissected cortices were triturated in DMEM (Sigma-Aldrich, Oakville, ON, Canada). The cell suspension was passed through a 70 μm cell filter strainer and then seeded into flasks (2 cortices/T75 flask). Culture media (DMEM supplemented with 10% FBS, 10 units/mL penicillin, and 10 μg/mL streptomycin) was replaced 3 days after plating and every second day thereafter. Primary astrocytes reached subconfluence at 7–8 days in vitro. Subconfluent cells were vigorously shaken to remove cells loosely attached to the astrocyte monolayer (mainly oligodendrocytes and microglia). Astrocytes were then harvested with trypsin-EDTA (Invitrogen Surrey, BC, Canada) and frozen in freezing medium (90% FBS, and 10% DMSO). Frozen astrocytes were thawed and plated on culture dishes. Cultures were maintained for 5–7 days prior to experiments. All experiments were carried out on confluent astrocytes and performed independently at least three times. Astrocytes isolated from different breeding pairs were used for each set of experiments [39].

### 2.5. In Vitro Scrape Loading Dye-Transfer Assay

The scrape-loading assay allows the introduction of a GJ-permeant fluorescent dye, such as Lucifer Yellow CH (LY) into cells to monitor its propagation into GJ-coupled cells within minutes after loading. Astrocytes were gently washed in PBS and then incubated at 37 °C for 20 min to increasing concentrations of danegaptide (0.01, 0.1, 1.0 or 10.0 µg/mL) in 1 mL of PBS. Dye entry was induced by scraping a confluent monolayer of astrocytes, incubated with 50 µL of the GJ permeable dye LY (0.5%) and GJ impermeable dye dextran-rhodamine (0.05%; 10 kDa) in PBS, with a scalpel blade and allowing the dyes to incubate for 2 min. Excess dye was washed off with several rinses of PBS. The extent of coupling was determined by epifluorescence Zeiss Axioplan2 fluorescence microscope measuring the area (in pixels) of LY diffusion subtracted by the area of the GJ impermeable reference dye, dextran rhodamine, with ImageJ software [40].

### 2.6. Western Blot Analysis

Protein samples were isolated from separate cultures of astrocytes incubated with either 0.01, 0.1, 1.0 or 10.0 µg/mL of danegaptide in 1 mL of PBS and resolved by sodium dodecyl sulfate-polyacrylamide gel electrophoresis as described previously [41]. Briefly, 40 μg of protein was separated on a 12% sodium dodecyl sulfate–polyacrylamide gel and transferred to polyvinylidene difluoride membranes. Membranes were processed and incubated overnight at 4 °C with primary antibodies against Cx43 (1:5000; catalog number: C6219; Sigma-Aldrich, Toronto, ON, Canada); mouse anti-γ-tubulin (1:3000 catalog number: T6557; Sigma-Aldrich, Canada), in Tris-buffered saline (TBS; Tris 50 mM, NaCl 150 mM, pH 8.0) containing 0.05% Tween (TBST) in 1% non-fat dry milk. The membranes were washed and incubated with horseradish peroxidase-conjugated secondary antibody (Vector Laboratories Inc, Burlingame, CA, USA) 1/5000 in TBST containing 5% non-fat dry milk. Immunoreactive proteins were visualized by chemiluminescent solution (Super Signal West Pico, Pierce Biotechnology Inc, Surrey, BC, USA).

### 2.7. Hemichannel Assay

Time-lapse fluorescence imaging to measure hemichannel activity was performed as previously described [40]. Briefly, WT astrocytes in cell culture dishes were gently washed twice with Locke’s solution containing (in mM) 154 NaCl, 5.4 KCl, 2.3 CaCl2, and 5 HEPES, at pH 7.4 and incubated in either 5 mM ethidium bromide (EtBr) or 5 mM EtBr and 1 µg/mL danegaptide. Fluorescence intensity was recorded every 30 s for 15 min with a Zeiss Axioplan2 fluorescence microscope. To induce hemichannel activity, control cells were incubated in Ca^2+^/Mg^2+^-free solution and fluorescence intensity was measured every 30 s for 15 min. Images of ethidium uptake were analyzed with the ImageJ software. For quantification, region of interest (ROI) was selected for at least 200 nuclei and ROI was selected for background (no cells). Fluorescence for each ROI at each time point 0 to 10 min was measured. Then, data was exported to Excel and background was subtracted for each ROI. Data was then exported to Graphpad software for statistical analysis.

### 2.8. Statistics

Animals for surgery and cell isolation were randomly selected and experimenter blinded to the genotype and treatments. A one-way ANOVA (one factor) was used to compare multiple means. When appropriate, post-hoc comparisons were made (specified in figure legends). Unpaired t-tests were performed when comparing between two groups. All *p*-values ≤0.05 were considered statistically significant. Statistics was performed using Prism 6 (Graph Pad, San Diego, CA, USA).

## 3. Results

### 3.1. Concentration-Dependent Increase in Cx43 Dye Coupling in Astrocytes Exposed to Danegaptide

**Figure 1:**
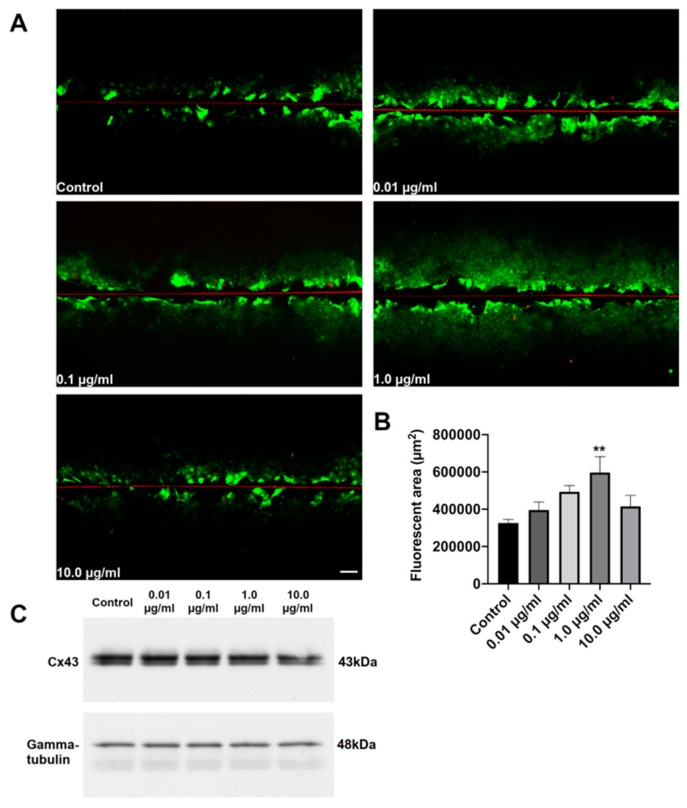
(**A**) In vitro scrape-loading assay (red line shows scrape edge) showing WT astrocytes exposed to 0.01, 0.1, 1.0 or 10.0 µg/mL danegaptide for 20 min followed by incubation with LY (green) and dextran rhodamine (red). Scale bar = 50 μm. (**B**) Quantification of total average diffusion area in LY pixels normalized to dextran rhodamine pixels in WT astrocytes subjected to either 0.01, 0.1, 1.0 or 10.0 µg/mL danegaptide for 20 min. One-way ANOVA followed by Dunnett’s multiple comparisons test, ** *p* = 0.0097; n = 4. Error bars represent mean ± SEM. (**C**) Representative immunoblot of Cx43 (upper blot) and γ-tubulin (lower blot) from WT astrocytes exposed to either 0.01, 0.1, 1.0 or 10.0 µg/mL danegaptide for 20 min, n = 3 independent experiments.

Danegaptide is a dipeptide chemically derived from the class of antiarrhythmic peptides such as rotigaptide [28]. Similarly to rotigaptide, danegaptide has been reported to maintain Cx43 GJ coupling, probably by functional modifications of Cx43 that reduce GJ closing and prevent intercellular uncoupling [32,42]. To determine whether danegaptide exhibits a similar effect on reducing GJ closing in astrocytes, we incubated astrocytes with increasing concentrations (0.01, 0.1, 1.0 or 10.0 µg/mL) of danegaptide in 1 mL PBS. A concentration-dependent increase in dye coupling was observed with a significant (*p* = 0.0097) 1.8-fold increase at 1 µg of the drug (Figure 1A,B). However, at 10 µg, the level of dye coupling was similar to control levels (Figure 1A,B).

To determine whether danegaptide had an effect on Cx43 expression, astrocytes were similarly incubated with increasing concentrations of the drug and subjected to Western blot. There were no marked changes in Cx43 protein levels in astrocytes exposed to either 0.01, 0.1, 1.0 or 10.0 µg/mL of danegaptide (Figure 1C). Collectively, these results are similar to what was previously reported for the antiarrhythmic peptide rotigaptide in cardiac myocytes and HeLa cells [31].

### 3.2. Danegaptide Does Not Affect Hemichannel Activity in Astrocytes In Vitro

**Figure 2:**
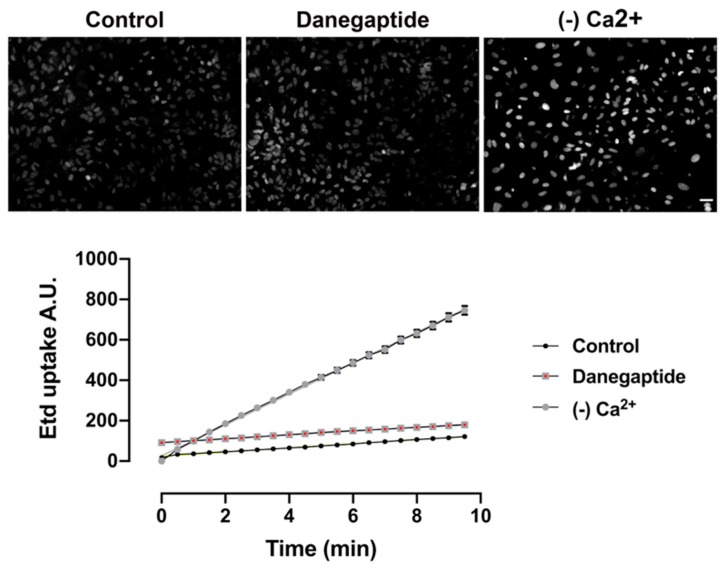
Micrographs above show examples of EtBr uptake after 10 min in WT astrocytes exposed to either PBS, 1 µg/mL danegaptide or Ca^2+^/Mg^2+^-free solution (divalent free) to induce hemichannel opening after 10 min incubation. Scale bar = 100 µm Graph below shows representative time-lapse measurements of ethidium uptake in WT astrocytes exposed to 5 μM EtBr with either control (PBS), 1 µg/mL danegaptide or Ca^2+^/Mg^2+^-free solution (divalent free) to induce hemichannel opening. Linear regression performed in control and danegaptide reveals slopes are not significantly different (n = 3 independent experiments).

In addition to the coupling function of Cx43, the occurrence of functional hemichannels (i.e., hemichannels that can be turned into the open state) composed of Cx43 was demonstrated in primary cultures of astrocytes in the absence of external Ca^2+^ [14,43]. To determine whether Cx43 hemichannel activity is affected by danegaptide in astrocytes, dye uptake assay was performed on astrocytes in PBS. Decreasing extracellular divalent cations is a well-known stimulus for hemichannel opening; in line with this, we found that applying Ca^2+^- and Mg^2+^-free solution, on our positive control astrocytes, triggered ethidium uptake in astrocytes (Figure 2). In contrast to the coupling effects of danegaptide, hemichannel activity was not affected when astrocytes were exposed to 1 µg of the drug in PBS (Figure 2).

### 3.3. MALDI IMS Was Able to Detect the Presence of Danegaptide in Brain Tissue

**Figure 3:**
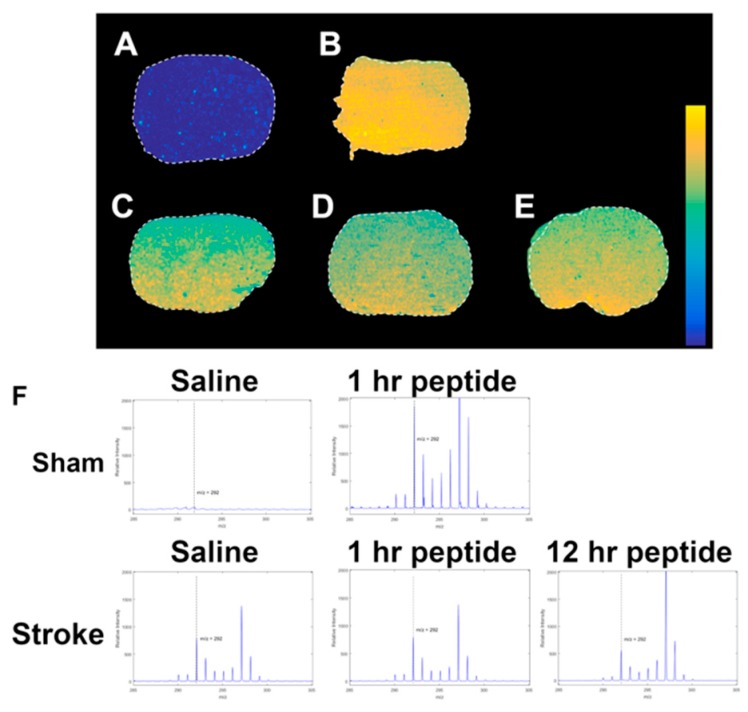
To assess danegaptide levels in the brain, 10 μm-thick coronal brain sections were thaw mounted onto ITO glass slides and processed for imaging mass spectrometry in positive reflectron mode on the 5800 TOF/TOF ABSciex instrument. Using mass spectrometry imaging-induced ion images, the spatial distribution of danegaptide was observed using image comparison. (**A**) Sham saline injected 1 h before tissue removal. (**B**) Sham peptide injected 1 h before tissue removal. (**C**) tMCAO 12 h saline. (**D**) tMCAO 1 h peptide and (**E**) tMCAO 12 h peptide. (**F**) Spectral comparison throughout the brain tissue sectioned from mice injected with the peptide, consistent with passage through the blood brain barrier. After 50 min of ischemia, saline or 75 µg/kg of danegaptide was injected intravenously. After reperfusion, 300 µg/kg of danegaptide was delivered by IP at 1, 2, and 3 h after the initial injection. Pseudo colour scale: blue low levels and yellow high level of signal. (n = 5).

MALDI IMS analysis of intact tissue sections was used to determine whether danegaptide penetrated the brain parenchyma under ischemic conditions. A significant increase in signal intensity was observed for images of danegaptide-injected samples in comparison to those injected with saline (Figure 3A). These samples were extracted 1 h following IP injection of either saline or danegaptide solutions. Alternatively, mobility of danegaptide in ischemic conditions was also investigated using MALDI IMS. Samples were treated with tMCAO followed by reperfusion. Either saline or danegaptide solutions were injected 10 min prior to reperfusion for these samples. Based on the images obtained, signal of greater intensity could be seen permeating the entire tissue section for tissue samples injected with danegaptide in comparison to saline-injected samples (Figure 3A). This was further supported by the MS spectra of the tissue region (Figure 3B). The increase in signal intensity indicates successful transport of danegaptide even under ischemic conditions. This is consistent with a recent study showing that danegaptide crosses the BBB, under different stroke models, when IP injected [44].

### 3.4. Danegaptide Reduced Infarct Volume in Mice Subjected to Ischemia/Reperfusion

Danegaptide is a small orally available therapeutic peptide that exhibits protective characteristics under ischemia/reperfusion injury, in different tissue types [30,45]. To investigate whether danegaptide could be protective in stroke, mice were therapeutically treated with the drug under an ischemia/reperfusion stroke model tMCAO.

**Figure 4:**
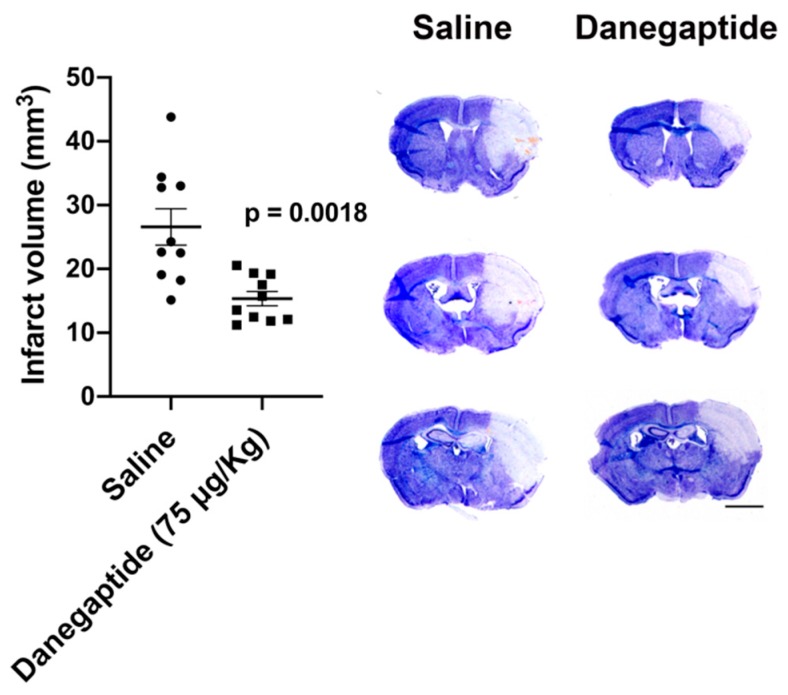
Quantification of infarct volume from WT (C57Bl/6) mice 48 h after tMCAO treated with either saline or 75 µg/kg danegaptide after 50 min of ischemia, intravenously. After reperfusion, 300 µg/kg of danegaptide was delivered by IP at 1, 2, and 3 h after the initial injection. (unpaired t-test; saline: n = 10 mice; danegaptide: n = 10 mice). Photomicrographs of thionin-stained sections from saline and danegaptide-treated mice. Pale blue region indicates infarct tissue. Scale bar = 2 mm.

Brain ischemia was induced in mice by occluding the CCA and MCA. After 50 min of ischemia, saline or 75 µg/kg [30] of danegaptide was administered via tail vein injection. After 60 min of ischemia, reperfusion was introduced and mice were treated with subsequent danegaptide at 1, 2 and 3 h after the initial injection. Mice were allowed to recover for 48 h and infarct volume was assessed. A significant (*p* = 0.0018) 42.2% reduction in infarct volume was measured in those mice treated with danegaptide, compared to controls (Figure 4).

In a small cohort of animals, we tested whether danegaptide, at increasing concentrations (1, 6.5, or 10 mg/kg), would have a similar protective effect in mice subjected to pMCAO. In contrast to the effects observed in tMCAO study, danegaptide did not provide significant protection in a pMCAO stroke model (Supplementary Figure S3).

## 4. Discussion

Present findings indicate that danegaptide promotes astrocytic Cx43 coupling, while having no effect on Cx43 hemichannel activity under basal in vitro conditions. Danegaptide was also detected in the brain parenchyma, indicating BBB permeability of this peptide. Furthermore, pharmacological use of danegaptide in tMCAO was neuroprotective at 48 h after the stroke event (other time points were not examined in this study). Taken together, this study underscores danegaptide as a potential therapeutic against tMCAO and sets the stage for further study of this drug in the context of stroke.

Danegaptide was developed as a dipeptide with similar characteristics to those of rotigaptide, that is, enhancing Cx43 GJ conductance in order to decrease ischemia-induced arrhythmias [46]. A recent study demonstrated that retinal vascular cell treated with danegaptide preserved Cx43 GJ intercellular communication, decreased cell death, and reduced cell monolayer permeability, in an in vitro model of diabetic retinopathy [32]. In our study, we show that under normal culture conditions, danegaptide promoted a concentration-dependent increase in astrocyte Cx43 GJ coupling. However, at higher concentrations (10 µg/mL), the GJ coupling effect of danegaptide was diminished. Similarly, Kim et al. (2018) showed that rat retinal endothelial cells grown in high glucose resulted in Cx43 uncoupling; however, at 100 nM, danegaptide significantly increased Cx43 coupling [32]. Consistent with our results, at higher concentrations of danegaptide, the coupling effect was lost in rat retinal endothelial cells grown in high glucose [32]. We showed that these changes in coupling were not due to danegaptide-dependent effects on Cx43 expression. Similarly, others have shown that rotigaptide does not affect Cx43 protein levels in cardiac myocytes and HeLa cells expressing Cx43 [31].

While danegaptide increase Cx43 GJ communication between astrocytes, under normal culture conditions, surprisingly, danegaptide does not affect Cx43 hemichannel activity. By contrast, others have also shown that danegaptide reduces dye uptake in cultured C6 glioma cells [28]. The different effects of danegaptide on Cx43 GJ and hemichannels could be due to the sensitivity of the drug to the conformational changes of Cx43 between the GJ and hemichannel state. Whether danegaptide affects Cx43 hemichannels under pathological conditions is an important question to be addressed in future studies.

A growing number of therapeutic targets against connexins are being developed [47]. However, delivery of pharmacological agents in the brain are a challenge due to the tight BBB. A previous study demonstrated that IP-injected danegaptide crosses the BBB and was clearly detected in ischemic mouse brain tissue [44]. Similarly, using MALDI-IMS techniques, we observed danegaptide in brain parenchyma in mice subjected to ischemia/reperfusion. More importantly, we show here that administration of danegaptide during tMCAO is neuroprotective. In contrast, danegaptide did not significantly decrease infarct volume in mice subjected to pMCAO. These contrasting results may reflect Cx43 behavior under different stroke models. For example, Martínez et al. (2000) demonstrated that rat cortical astrocytes subjected to 12 h hypoxia exhibited Cx43 dye coupling similar to controls. However, 15–30 min after reoxygenation, dye coupling was transiently reduced by approximately 70%. The reduction in dye coupling occurred without changes in the levels of Cx43 [48]. Others have shown similar effects of astrocytic Cx43 coupling in hypoxia/reoxygenation experiments [25]. It is possible that in the pMCAO model, Cx43 gap junctions remain coupled and, therefore, danegaptide provides little effect. We recently reported that Cx43 coupling increases 2 h after pMCAO [26]. In the tMCAO model, reperfusion, which is similar to reoxygenation, results in increased Cx43 GJ uncoupling; in this situation, danegaptide would promote Cx43 GJ stabilization and coupling between astrocytes. The increased danegaptide-dependent coupling between astrocytes may buffer the detrimental effects of reperfusion; namely, generation of both reactive oxygen species and proinflammatory factors. Both of these processes have been reported to reduce Cx43 intercellular communication [21,39].

While the results of this study are promising and are consistent with other ischemia/reperfusion experiments in other organ systems [29,30,45,46], the detailed mechanism of action of danegaptide is not fully elucidated [46] and requires further study

## 5. Conclusions

The findings presented here highlight danegaptide as a potential therapeutic peptide specifically for an ischemia/reperfusion model of stroke. Manipulating astrocytic networks by pharmacologically targeting GJ channels may provide a novel strategy for therapeutic intervention. It is the goal of this study to stimulate future research regarding this drug in the context of stroke.

## Supplementary Materials

The following are available online at https://www.mdpi.com/2218-273X/10/3/353/s1, Figure S1: Diagram indicating method used to induce tMCAO. First, a 70-g clamp is applied to carotid (1). Second, a pin is inserted under MCA causing stroke (2). The pin (2) is tapered allowing for reducing blockage by reducing pressure via shifting the pin slowly away from MCA, Figure S2: Heat map of blood flow during tMCAO left panels. A reduction in blood flow is observed when clamp is applied to carotid. After pin is applied under MCA, indicated by black arrow (clearly seen in black and white image middle panels) blood flow is further reduced. Pin is slowly removed (10 min and 20 min) showing a slow return of blood flow. Once clamp is removed from carotid, a considerable increase in blood flow was observed at reperfusion. Blue colour represents low or no flow of blood. Right panel show coloured photos of surgical procedure, Figure S3: Photomicrographs of thionin-stained sections 48 h after pMCAO in WT mice treated with either saline, 1 mg/kg, 6.5 mg/kg or 10 mg/kg danegaptide, pale blue area indicates the infarct delineated by black outline. Scale bar = 2 mm. Graph below indicates infarct volume 48 h after pMCAO from WT mice treated either saline, 1 mg/kg, 6.5 mg/kg or 10 mg/kg danegaptide (one-way ANOVA followed by Dunnett’s multiple comparisons test; saline versus scrambled: saline vs. danegaptide (1 mg/kg), *p* = 0.8639; saline vs. danegaptide (6.5 mg/kg), *p* = 0.7579; saline vs. danegaptide (10.0 mg/kg) *p* = 0.1528; saline: n = 6 mice; danegaptide (1 mg/kg): n = 3 mice; danegaptide (6.5 mg/kg): n = 3 mice; danegaptide (10.0 mg/kg): n = 3). Error bars represent mean ± SEM.
