# Supplementary material for: Danegaptide Enhances Astrocyte Gap Junctional Coupling and Reduces Ischemic Reperfusion Brain Injury in Mice
Source: Biomolecules. 2020 Feb 26;10(3):353. doi: 10.3390/biom10030353 (PMC7175267; doi:10.3390/biom10030353)

Appendix A

Supplementary Figure 1. Diagram indicating method used to induce tMCAO. First, a 70-gram clamp is applied to carotid, (1). Second, a pin is inserted under MCA causing stroke (2), The pin (2) is tapered allowing for reducing blockage by reducing pressure via shifting the pin slowly away from MCA.

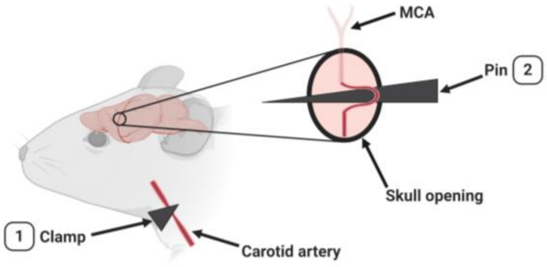

Supplementary Figure 2. Heat map of blood flow during tMCAO left panels. A reduction in blood flow is observed when clamp is applied to carotid. After pin is applied under MCA, indicated by black arrow (clearly seen in black and white image middle panels) blood flow is further reduced. Pin is slowly removed (10 min and 20 min) showing a slow return of blood flow. Once clamp is removed from carotid, a considerable increase in blood flow was observed at reperfusion. Blue colour represents low or no flow of blood. Right panel show coloured photos of surgical procedure.

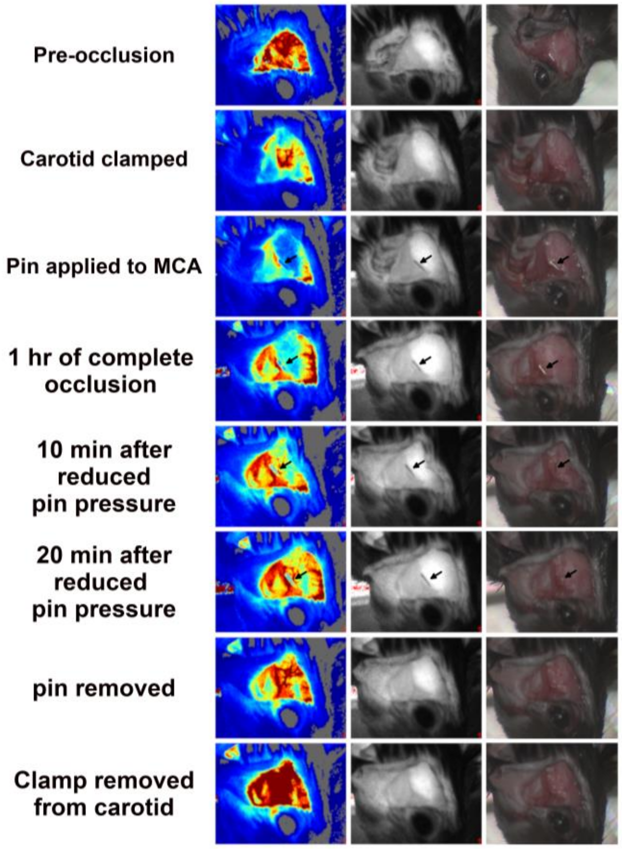

Supplementary Figure 3. Photomicrographs of thionin-stained sections 48 after pMCAO in WT mice treated with either saline, 1 mg/Kg, 6.5 mg/Kg or 10 mg/Kg danegaptide, pale blue area indicate the infarct. Scale bar = 2 mm. Graph below indicate Infarct volume 48 hrs after pMCAO from WT mice treated either saline, 1 μg/g, 6.5 μg/g or 10 μg/g danegaptide (one-way ANOVA followed by Dunnett's multiple comparisons test; saline versus scrambled: Saline vs. Danegaptide (1 μg/g), $p = 0.8639$; Saline vs. Danegaptide (6.5 μg/g), $p = 0.7579$; Saline vs. Danegaptide (10.0 μg/g) $p = 0.1528$; saline: $n = 6$ mice; Danegaptide (1 μg/g): $n = 3$ mice; Danegaptide (6.5 μg/g): $n = 3$ mice; Danegaptide (10.0 μg/g): $n = 3$). Error bars represent mean ± SEM.

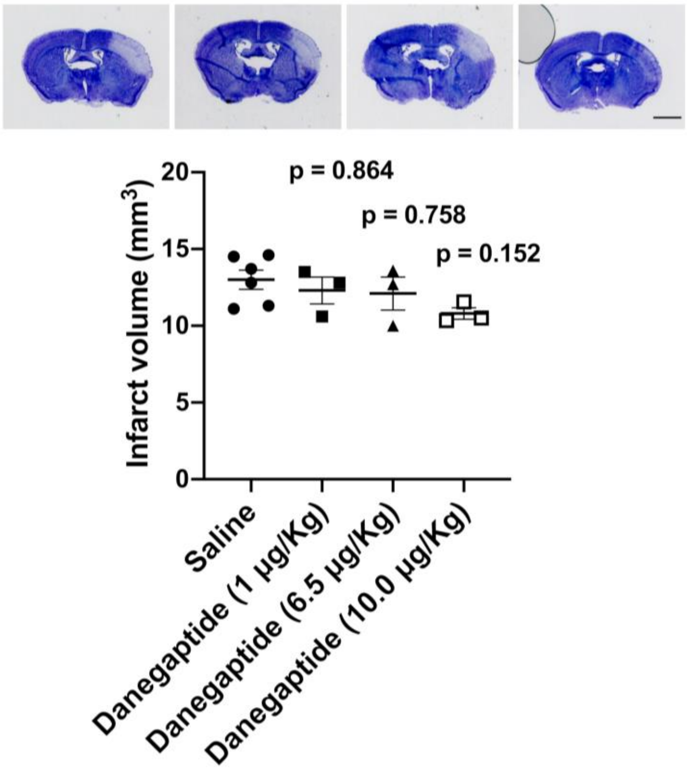

Supplement: Supplementary file 1 [file biomolecules-10-00353-s001.pdf]
